# Supplementary material for: Automated Adenoid Hypertrophy Assessment with Lateral Cephalometry in Children Based on Artificial Intelligence
Source: Diagnostics (Basel). 2021 Jul 31;11(8):1386. doi: 10.3390/diagnostics11081386 (PMC8394806; doi:10.3390/diagnostics11081386)
Supplement: Supplementary file 1 [file diagnostics-11-01386-s001.zip › diagnostics-1299014-SI.pdf]

Automated Adenoid Hypertrophy Diagnosis with Lateral Cephalometry based on Artificial Intelligence

Supplementary File

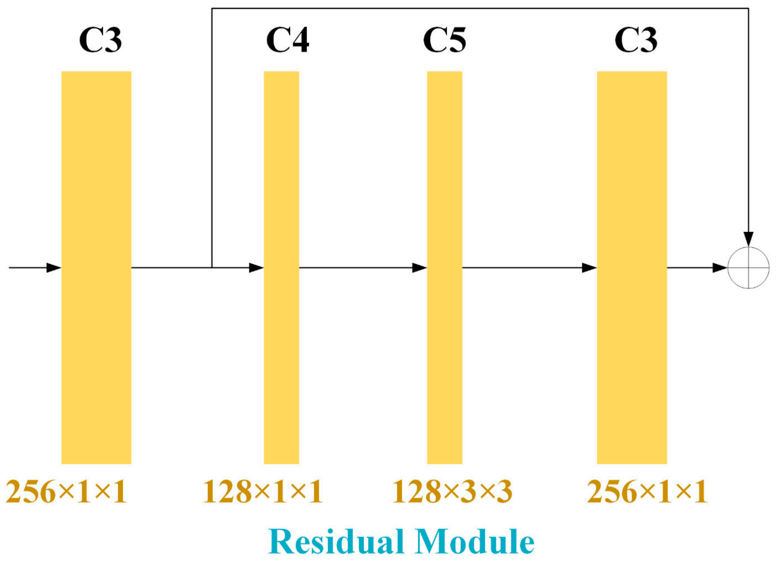

Supplementary Figure S1. Residual Module as Building Block used in Hourglass.

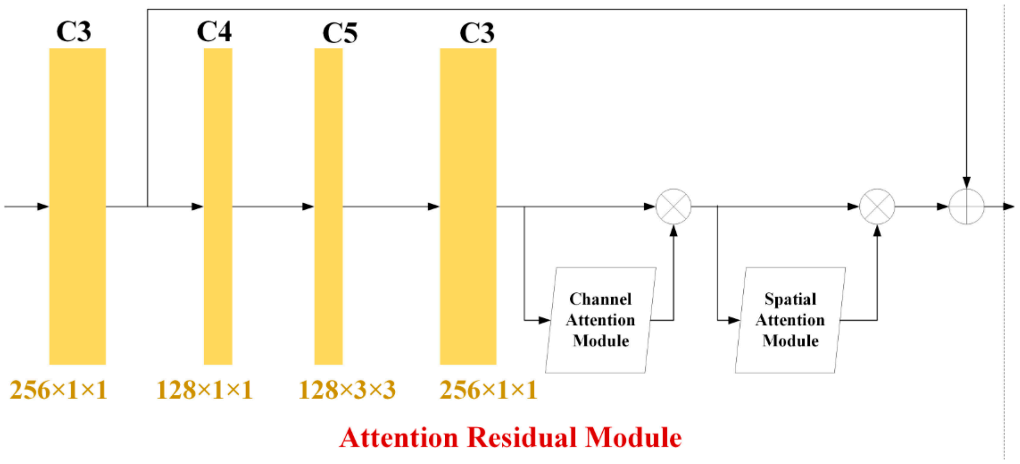

Supplementary Figure S2. Residual Block with Channel Attention Module and Spatial Attention Module.

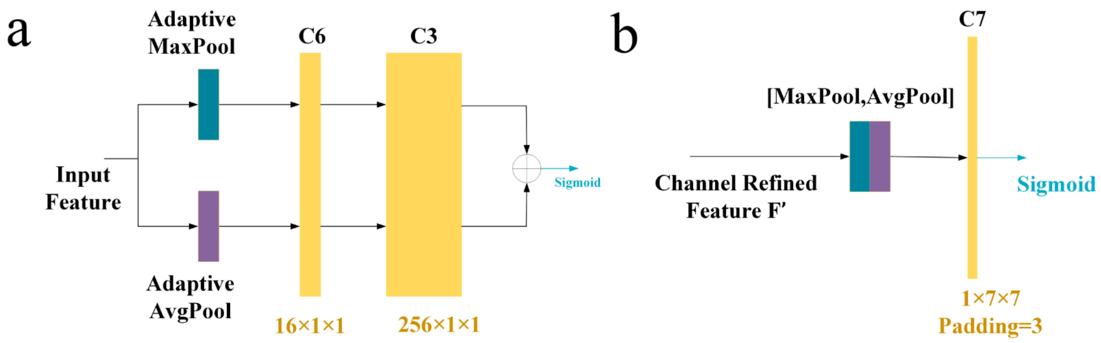

Supplementary Figure S3. The Channel Attention Module (a); Spatial Attention Module (b).

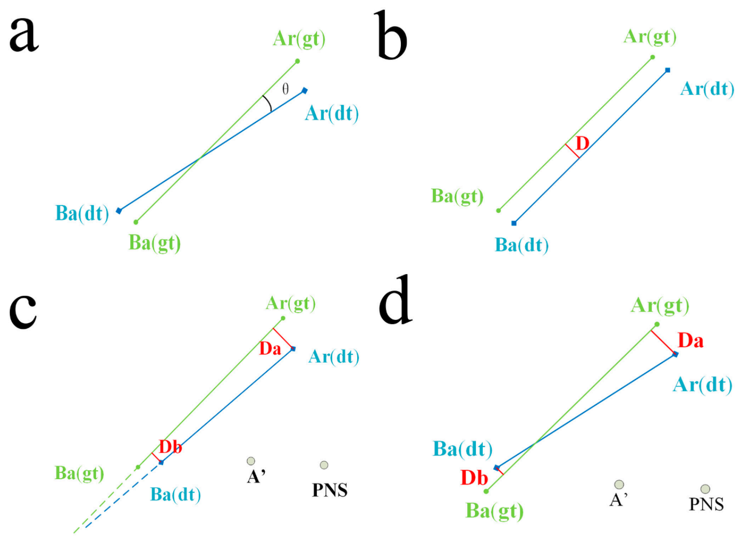

Supplementary Figure S4. Ideal Rotation between Ground Truth (gt) and Detection (dt) (a); Ideal Translation between Ground Truth and Detection (b); Real Rotation and Translation Case (c and d).
